# Bilateral neck cysts as an isolated sonographic finding in the antenatal detection of fetal aneuploidy: a case report

**DOI:** 10.4076/1757-1626-2-8322

**Published:** 2009-07-06

**Authors:** Khalil Abi-Nader, Elisa Filippi, Pranav P Pandya, Elisabeth Peregrine

**Affiliations:** 1 Fetal Medicine Unit, The Elizabeth Garrett Anderson Obstetric Hospital, Institute for Women's Health, University College London Hospitals NHS Trust, London, UK

## Abstract

Isolated fetal lateral neck cysts can represent a cystic hygroma or a developmental remnant cyst. In the absence of an increased nuchal translucency or associated malformations the risk of aneuploidy has been considered negligible. Still, dysmorphology in aneuploid fetuses might not be evident except at a later stage. We report on a case of isolated fetal bilateral neck cysts where aneuploidy was suspected and confirmed despite the lack of associated morphologic abnormalities.

## Case presentation

A 34 year old middle eastern lady with a spontaneous conception presented at 13 weeks of gestation for a routine 1^st^ trimester ultrasound and screening. Her booking weight was 79 kg and her height 165 cm. She was a housewife with three previous uncomplicated pregnancies delivered vaginally. She was on no medication except for iron supplement. The past medical and surgical history was unremarkable and the patient was neither a smoker nor an alcohol drinker. The family history of her partner and herself was unremarkable as well.

**Figure 1:**
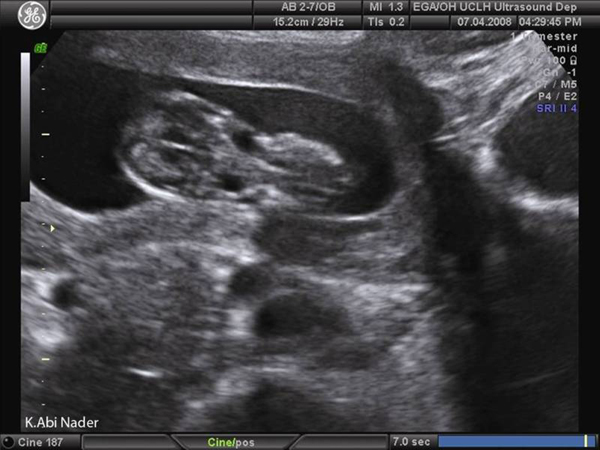
**Coronal section: A coronal ultrasound section of the fetal head and neck region at 13 weeks of gestation shows bilateral cysts in the middle-half of the neck**.

**Figure 2:**
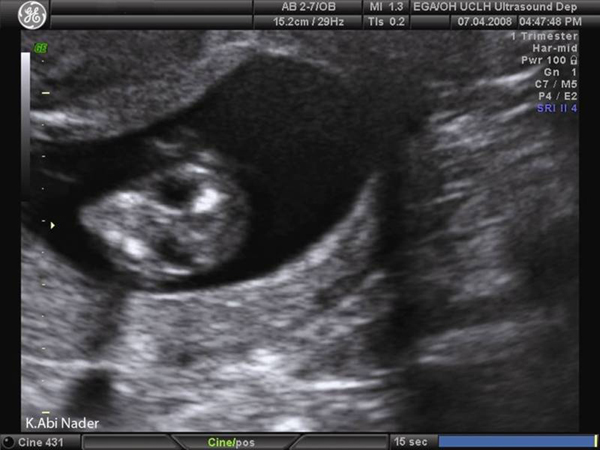
**Trans-axial section: The same cysts seen in Figure **1**are shown here in cross section using ultrasound.** The cysts are located anterolaterally in the neck and the posterior nuchal region looks normal.

On 1^st^ trimester ultrasound, the fetus was noted to have bilateral anterolateral neck cysts around 5 × 5 mm in size (Figures 1 and 2) suggestive of either 'non-septated cystic hygromas' which are congenital malformations of the lymphatic system characterized by fluid-filled jugular lymphatic sacs of the fetal neck [1,2] or, the much rarer bilateral branchial cleft cysts. No other abnormalities were noted and the nuchal translucency measured 2.3 mm. The patient was counselled for the high possibility of spontaneous regression and good fetal outcome awaiting the results of the integrated 1^st^ trimester screening test. At 16 weeks, integrated screening gave a 1 in 2 risk for trisomy 18 and therefore fetal ultrasonograpy was repeated. The fetal growth and amniotic fluid volume were normal and the bilateral neck cysts were stable in size. No other abnormalities could be detected despite a careful sonographic assessment and an amniocentesis for fetal karyotyping was offered and accepted. PCR on the amniotic fluid revealed trisomy 18 which was confirmed by cytogenetic analysis.

**Figure 3:**
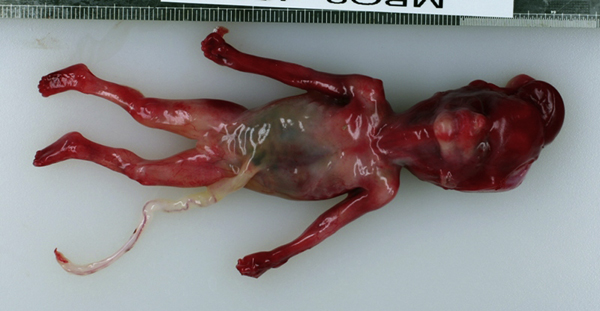
** Post-mortem appearance of the fetus: The external findings are low-set ears, legs fixed in extension and clenched fists**. The scalp was damaged during the delivery.

The couple decided to opt for termination of pregnancy which was completed medically at 17+4 weeks with no complications. Pathologic examination of the fetus revealed a dysmorphic face with low-set ears, both legs fixed in extension and clenched fists. At autopsy the neck cysts could not be identified, but there had been considerable trauma to the scalp during delivery. Internally the kidneys and adrenals were of normal size and shape but there was dysplasia on microscopy. There was abnormal placental villous morphology, of a pattern consistent with aneuploidy. There were no internal gross structural anomalies that had been missed on the scan.

## Discussion

The differential diagnosis of fetal anterolateral neck cysts includes dilated jugular lymphatics defining a cystic hygroma [1], the presence of branchial cleft cysts, and a thyroid cyst [3]. Non-septated cystic hygromas are unilateral or bilateral, simple appearing cysts that are located in the antero-lateral cervical region [4]. Bilateral branchial cleft cysts are extremely rare representing only 1% of branchial cleft cysts [5] and antenatal diagnosis is barely reported for the unilateral cases [6]. Thyroid cysts on the other hand, usually occur in association with maternal thyrotoxicosis [3].

Antenatally detected isolated bilateral neck cysts without an increase in nuchal translucency have been linked to physiologic delay in jugular lymphatic development leading to spontaneous resolution before 17 weeks of gestation [2]. While Bronstein et al. noted a 5% aneuploidy rate in the presence of this finding, cases with trisomy 18 or 21 had additional associated abnormalities [7]. Other investigators did not find an association between fetal aneuploidy and islolated lateral neck cysts in the absence of an increased nuchal translucency [1,2,8].

This case clearly indicates that the presence of isolated fetal lateral neck cysts in the first trimester and early second trimester should not be dismissed directly as a variant of normal development. Close follow up should be instituted as structural abnormalities may become apparent at a slightly later gestation [9]. Invasive testing for fetal karyotype should be considered. If the neck cysts resolve before mid-gestation and no additional abnormalities are detected on subsequent scans, the outlook for the pregnancy is excellent [7].

## Abbreviation

PCR: Polymerase chain reaction.

## Consent

Written informed consent was obtained from the patient for publication of this case report and accompanying images. A copy of the written consent form is available for review of the Editor-in-Chief of this journal.

## Competing interests

The authors declare they don't have any competing interests.

## Authors' contributions

KNAN acquired and interpreted fetal ultrasound findings, laboratory screening and genetic results and was the major contributor in writing the manuscript. EF was involved in the manuscript's design and gave a major contribution towards the literature search on prenatal diagnosis of fetal neck cysts. EP was involved in drafting the manuscript and reviewed critically the case and the content of the discussion. All the authors read and approved the final manuscript.
